# The epidemiology of fecal carriage of nontyphoidal *Salmonella* among healthy children and adults in three sites in Kenya

**DOI:** 10.1371/journal.pntd.0011716

**Published:** 2023-10-26

**Authors:** Esther M. Muthumbi, Alfred Mwanzu, Cecilia Mbae, Godfrey Bigogo, Angela Karani, Salim Mwarumba, Jennifer R. Verani, Samuel Kariuki, J. Anthony G. Scott

**Affiliations:** 1 Kenya Medical Research Institute–Centre for Geographic Medicine Research, Coast, Kilifi, Kenya; 2 Department of Infectious Disease Epidemiology, London School of Hygiene and Tropical Medicine, London, United Kingdom; 3 Kenya Medical Research Institute–Centre for Microbiology Research, Nairobi, Kenya; 4 Kenya Medical Research Institute–Centre for Global Health Research, Kisumu, Kenya; 5 U.S. Centers for Disease Control and Prevention, Division of Global Health Protection, Nairobi, Kenya; Menzies School of Health Research, AUSTRALIA

## Abstract

**Background:**

Despite the importance of non-Typhoidal *Salmonella* (NTS) disease in Africa, epidemiologic data on carriage and transmission are few. These data are important to understand the transmission of NTS in Africa and to design control strategies.

**Method:**

To estimate the prevalence of stool carriage of NTS in Kenya, we conducted a cross-sectional study in Kilifi, Nairobi, and Siaya, sites with a low, moderate and high incidence of invasive NTS disease, respectively. At each site, we randomly selected 100 participants in each age-group of 0–11 months, 12–59 months, 5–14 years, 15–54 years and ≥55 years. We collected stool, venous blood (for hemoglobin and malaria rapid tests), anthropometric measurements, and administered a questionnaire on Water Access Sanitation and Hygiene (WASH) practices. Stool samples were cultured on selective agar for *Salmonella*; suspect isolates underwent serotyping and antimicrobial susceptibility testing.

**Result:**

Overall, 53 (3.5%) isolates of NTS were cultured from 1497 samples. Age-adjusted prevalence was 13.1% (95%CI 8.8–17.4) in Kilifi, 0.4% (95%CI 0–1.3) in Nairobi, and 0.9% (95%CI 0–2.0) in Siaya. Prevalence was highest among those aged 15–54 years (6.2%). Of 53 isolates; 5 were *S*. Enteritidis, 1 was *S*. Typhimurium. No *S*. Typhi was isolated. None of the risk factors were associated with carriage of NTS. All isolates were susceptible to all antibiotics tested, including ampicillin, chloramphenicol, ciprofloxacin and co-trimoxazole.

**Conclusion:**

Prevalence of fecal carriage was high in Kilifi, an area of low incidence of invasive NTS disease and was low in areas of higher incidence in Nairobi and Siaya. The age-prevalence, risk factors, geographical and serotype distribution of NTS in carriage differs from invasive disease.

## Author summary

Infections with non-typhoidal *Salmonella* (NTS) bacteria can present as asymptomatic carriage, diarrhoea or invasive disease. Worldwide, invasive NTS disease (iNTS) is more common in Africa, especially among children, and is commonly caused by two serotypes: *S*. Typhimurium and *S*. Enteritidis. Recent studies suggest that transmission of these serotypes causing iNTS is human to human. Consequently, identifying the age-group and risk factors associated with asymptomatic carriage of NTS would help in controlling its transmission. We randomly sampled healthy children and adults across 3 sites in Kenya with carrying iNTS incidence. We found that carriage is highest among older children and adults, who can be targeted to reduce its transmission. We also found that carriage was highest in Kilifi, the area of low iNTS incidence, it was not associated with known risk factors for iNTS, and majority of the serotypes were neither *S*. Typhimurium nor *S*. Enteritidis. These differences between NTS carriage and invasive disease need further investigations to understand the linkages and inform control.

## Introduction

Transmission of non-typhoidal Salmonella (NTS) has been associated with contamination of food and with direct contact with chickens or reptiles [1–3]. These findings come from high-income countries where NTS disease is characterized by a self-limiting gastroenteritis, with an incidence of 634 per 100,000 cases per year in Europe and North America, and with a case fatality ratio (CFR) of 0.2% [4]. By contrast, NTS disease in Africa is more frequently invasive, causes little or no diarrhea, and has a higher CFR [5]. In 2017, the incidence of invasive NTS (iNTS) disease was 34.5 cases per 100,000 person-years, and CFR of 16% across sub-Saharan Africa [6].

The genotypes most commonly causing iNTS disease in Africa, *Salmonella* Typhimurium and Enteritidis, have no known animal reservoir [7]. Genetic analysis in Kenya has previously shown that *Salmonella* Typhimurium isolates obtained from index cases of NTS bacteremia in children and their human contacts are identical but are distinct from those found in their animal contacts [8]. Similar observations have been made in The Gambia [9] and Burkina Faso [10]. It has been postulated that transmission of iNTS disease in Africa is principally human to human [7]. This theory has been strengthened by the discovery of a novel sequence type of *Salmonella* Typhimurium, ST313, and a novel clade of *Salmonella* Enteritidis, ‘the African clade’, which are leading causes of iNTS in Africa and whose genomes have undergone changes in a pattern that is associated with host restriction, similar to *Salmonella* Typhi [11,12]. If the transmission is human-to-human, then the subgroup of the population that are carriers acts as an important reservoir for the organism and may be the main source of infection.

Fecal shedding, especially in settings with suboptimal hygiene and sanitation, could lead to high transmission rates driving a high risk of invasive disease. However, the epidemiology of carriage of NTS and its association with iNTS in Africa, are not well understood. We aimed to explore the epidemiology of carriage in Kenya by studying healthy individuals from three sites with different incidence of iNTS disease.

## Method

### Ethics statement

Written informed consent was obtained from all adult participants and from the guardians of children aged <18 years old. Assent was obtained from all participants aged 13–17 years old. The study was approved by the Kenya Medical Research Institute (KEMRI) Scientific and Ethics Research Unit (SERU No. 3221). This activity was reviewed by CDC and was conducted in a manner consistent with applicable federal law and CDC policy [Project ID: 0900f3eb81e92cdd].

### Study sites

The study sites were: Junju location in Kilifi County, Mukuru in Nairobi County, and Asembo location in Siaya County. Junju location is a rural setting with a population of approximately 34,000 people over 115 km^2^. [13] Mukuru is a densely populated urban slum area, with approximately 250,000 people in an area of 3 km^2^, Asembo is a sparsely populated rural location, with a population of approximately 25,000 people over 100 km^2^ in a defined surveillance catchment area [13,14]. The distribution of risk factors for iNTS disease differs by site. Malaria prevalence was estimated as 8%, 0.2% and 26% in Kilifi, Nairobi and Siaya [15], respectively, while HIV prevalence was estimated as 3%, 5% and 14%, respectively [16]. The incidence of iNTS disease in children under 5 years is estimated as 36–88 per 100,000 person-years in Kilifi and 501–3914 per 100,000 person-years in Siaya [17–19]. The incidence of iNTS in Mukuru slums has not been estimated previously, but can be inferred from that of neighboring Kibera slums as 255–998 per 100,000 person-years [19].

### Sampling framework

Sampling was done in October-November 2016 in Kilifi, May-September 2017 in Nairobi and June-September 2017 in Siaya. In Kilifi and Siaya, we used existing population registers as our sampling frame; these were available from the Kilifi Health and Demographic Surveillance System (KHDSS) [20] and from the Population-Based Infectious Disease Surveillance (PBIDS) [14] platform, which is nested within the KEMRI/CDC demographic surveillance system [21]. We undertook age-stratified, simple random sampling of the entire population register of the Junju and Asembo locations. To create a sampling frame in Mukuru, Nairobi, we used Google Earth, a GIS software (QGIS), and a high-resolution satellite image of the area. We generated random points in the study area and excluded points on schools, churches, and community halls when the points were identified in maps from Open Street Maps (http://openstreetmaps.org). At high resolution, the area had already been mapped into residential blocks by an ongoing Salmonella project [22]. We used random residential-block sampling followed by systematic age-stratified sampling. At each site we sampled 100 participants in each of five age strata: 1–11 months, 12–59 months, 5–14 years, 15–54 years, 55 years and above. Random lists of potential participants were created in each site in excess of the sample size targeted and any selected individuals who were absent or unwilling to participate (or any households in Nairobi that did not contain an age-appropriate participant) were replaced by selecting the next individual on the random list.

### Patient/Public Participation

Community Engagement with the public was performed before study recruitment. We held community *barazas* organised by the location chiefs, meetings with community representatives, and the Department of Health in Nairobi County and Kilifi County where we discussed the study. We highlighted the background of the study, study design, recruitment procedures, participants involvement, risks and benefits of the study. Peer reviewed research findings will be disseminated to participants and the participating communities upon completion of the study in dissemination meetings following the same channels.

### Interview and specimen collection

Following informed consent, study participants were given the choice to be interviewed at their home or in a local health clinic. A questionnaire was administered, and the participant was asked to provide a stool sample. The questionnaire inquired about demographics, sanitation practices, food and water consumption, ownership and contact with animals, clinical status in the preceding 2 weeks, and household crowding. For those interviewed at the clinic, a blood sample was taken for HRP2-based *P*. *falciparum* malaria rapid test and hemoglobin measurement (Mission strips). Anthropometric measures (height/length, weight and Mid-Upper Arm Circumference [MUAC] for children under 5 years) were also taken.

No samples were collected between 1^st^ and 23^rd^ August 2017 due to the Kenyan national elections. Those unable to produce a stool sample immediately were given a sterile pot for stool sampling at home the following morning and the sample was collected by study fieldworkers before noon. Both at home by the fieldworkers and at the clinic, an aliquot of the stool sample was inserted in Cary-Blair transport media for culture and preserved at 2–4°C in a cooler. Within 8 hours of collection, the stool samples were transported to a microbiology laboratory for processing.

Samples from Kilifi, Nairobi, and Siaya were processed in laboratories at KEMRI-Wellcome Trust Programme in Kilifi, KEMRI-Center for Microbiological Research in Nairobi and KEMRI Centre for Global Health Research- laboratories in Kisumu, respectively. The data collection tools and laboratory methods were standardized across all sites.

### Laboratory testing

In the laboratory, culture samples were incubated overnight in Selenite F broth (Oxoid microbiological products) for enrichment and sub-cultured onto selective media (Xylose Lysine Deoxycholate agar (XLD), Salmonella/Shigella agar (SS) agar and Brilliant Green media) to select for *Salmonella enterica* species. Species were confirmed using biochemical testing (API 20E). *Salmonella* Typhimurium and *Salmonella* Enteritidis were determined using the Kauffman-White scheme and commercial antisera. Antimicrobial susceptibility testing was performed and interpreted using Clinical Laboratory Standards Institute (CLSI) guidelines, for the following antimicrobials: ampicillin 10μg, amoxicillin/clavulanate 20/10 μg, cotrimoxazole 1.25/23.75μg, cefotaxime 10μg, chloramphenicol 10μg, ciprofloxacin 5μg, ceftazidime 30μg, ceftriaxone 30μg, imipenem 10μg, cefoxitin 30μg and amikacin 30μg [23].

### Data analysis

The prevalence of carriage in stool in each age stratum was estimated as the proportion of sampled individuals who had a positive stool culture and exact 95% confidence intervals were calculated. To account for the sampling scheme, total population prevalence for each site was age standardized using the age structure of the local population as population weights.

We defined underweight (weight-for-age Z score [WAZ] <-2); wasting (weight-for-height Z score [WHZ] <-2); and stunting (height-for-age Z score [HAZ] <-2) for children under 5 years using WHO Child Growth Standards [24]. Anemia was defined as hemoglobin <10g/dl, and recent malaria infection was defined as malaria HRP-2 rapid test positive. Risk factor analyses were conducted using Poisson regression with robust standard errors to estimate prevalence ratios. The outcome variable was presence of NTS in the stool samples. Variables associated with the outcome variable at a p<0.05 significance level were selected for inclusion in a multivariable regression model, including age, sex and site as *a priori* explanatory variables. Statistical analyses were performed using STATA v.13.

## Results

In Kilifi, 775 residents were selected for inclusion in the study, 506 were enrolled and 494 (response rate, 63.7%) were sampled. In Nairobi, 750 random points were generated by the software, 510 participants were enrolled and 504 (67.2%) were sampled. In Siaya, 607 residents were selected for the study, 506 were enrolled and 499 (82.2%) were sampled. In total, we sampled 1,497 participants; 703 (47%) were male (Table 1).

**Table 1:** Participant characteristics at enrollment, by site, Kenya (2016–17).

		Kilifi		Nairobi		Siaya	
		n/N	%	n/N	%	n/N	%
*Participant characteristics*							
Sex	Male	220/494	44.5	266/504	52.8	217/499	43.5
Pregnant, in females >18y		2/115	1.7	3/73	4.1	2/126	1.6
Recent malaria	Positive	23/307	7.5	11/443	2.5	139/489	28.4
Wasting, in < 5y	WHZ <-2	20/181	11.1	15/214	7.5	6/200	3
Stunting, in < 5y	HAZ <-2	50/198	25.3	43/214	20.1	42/200	21
Underweight, in < 5y	WAZ <-2	30/181	16.7	16/214	7.5	16/200	8
Acute Malnutrition, in < 5y	MUAC <12.5cm	34/197	17.3	54/212	25.5	8/200	4
Fever in last 2 weeks	Yes	61/458	13.3	47/486	9.7	152/499	30.5
Diarrhea in last 2 weeks	Yes	25/458	5.5	61/486	12.6	35/499	7
Admitted in last 2 weeks	Yes	7/458	1.5	11/486	2.3	7/499	1.4
*Household characteristics*							
Household member fever in last 2 weeks	Yes	44/458	9.6	26/486	5.4	156/499	31.3
Household member diarrhea in last 2 weeks	Yes	17/458	3.7	40/486	8.2	39/499	7.8
*WASH Practices*							
Source of drinking water	Piped water	80/458	17.5	4/486	0.8	18/499	3.6
	Public tap	236/458	51.5	482/486	99.2	214/499	42.8
	River/Lakes	1/458	0.2	0/486	0	77/499	15.4
	Well/Dams/Borehole	141/458	30.8	0/486	0	190/499	38.1
Uses soap for hand washing	Yes	351/458	76.6	471/486	96.9	463/499	92.8
Uses shared basin for washing hands	Yes	192/458	41.9	131/486	26.9	58/499	11.6
Toilet Type	Modern with/without flush	50/458	10.9	63/486	12.9	2/499	0.4
	Pit latrine	394/458	86.1	392/486	80.7	479/499	95.9
	None/Open defecation	10/458	2.2	1/486	0.2	10/499	2
	Other	4/458	0.1	30/486	6.2	8/499	1.6
Animal Ownership	Cattle	61/458	13.3	2/486	0.4	305/499	61.1
	Sheep	4/458	0.9	1/486	0.2	142/499	28.5
	Goats	129/458	28.2	8/486	1.7	194/499	38.9
	Pigs	1/458	0.2	1/486	0.2	3/499	0.6
	Chickens	256/458	55.9	57/486	11.7	452/499	90.6

Out of the 1,497 stool samples collected, 53 NTS isolates were recovered (crude prevalence 3.5%, 95% CI 2.6, 4.6%). Crude carriage prevalence was 9.3% (46/494) in Kilifi, 0.2% (1/504) in Nairobi and 1.2% (6/499) in Siaya. In Kilifi, the prevalence varied significantly by age (p = 0.035) and was low in infancy, highest in the 15–54 year age stratum at 17.4%, and lower in older age groups. The prevalence did not vary significantly by sex (p = 0.123, Table 2). When standardized using age-specific population weights at each site, the total population prevalence was 13.1%, 0.4%, 0.9% in Kilifi, Nairobi and Siaya, respectively.

**Table 2:** Age and sex-related prevalence of stool carriage of NTS in Kenya (2016–17).

	Kilifi			Nairobi			Siaya		
	n/N*	%	95% CI	n/N*	%	95% CI	n/N*	%	95% CI
*Female*									
0–11 m	3 / 55	5.5	1.1, 15.1	0 / 44	0		1 / 48	2.1	0,11.1
12–59 m	4 / 51	7.8	2.2, 18.8	0 / 50	0		0 / 62	0	
5–14 y	7 / 45	15.6	6.5, 29.5	0 / 72	0		0 / 51	0	
15–54 y	10 / 53	18.9	9.4, 31.9	1 / 57	1.8	0, 9.4	1 / 51	1.9	0,10.4
55+ y	6 / 70	8.6	3.2, 17.7	0 / 15	0		1 / 70	1.4	0, 7.7
Total	30 / 274	10.9	7.5, 15.3	1 / 238	0.4	0, 2.3	3 / 282	1.1	0.2, 3.1
*Male*									
0–11 m	5 / 71	7.0	2.3, 15.7	0 / 64	0		1 / 49	2	0, 10.9
12–59 m	2 / 43	4.7	0.6, 15.8	0 / 57	0		1 / 41	2.4	0, 12.9
5–14 y	3 / 40	7.5	1.6, 20.4	0 / 79	0		0 / 57	0	
15–54 y	5 / 33	15.2	5.1, 31.9	0 / 49	0		0 / 33	0	
55+ y	1 / 33	3.0	0, 15.8	0 / 17	0		1 / 37	2.7	0, 14.2
Total	16 / 220	7.3	4.2, 11.5	0 / 266	0		3 / 217	1.4	0.3, 4.0
*All*									
0–11 m	8 / 126	6.4	2.8, 12.1	0 / 108	0		2 / 97	2.1	0.3, 7.3
12–59 m	6 / 94	6.4	2.4, 13.4	0 / 107	0		1 / 103	0.9	0, 5.3
5–14 y	10 / 85	11.8	5.8, 20.6	0 / 151	0		0 / 108	0	
15–54 y	15 / 86	17.4	10.1, 27.1	1 / 106	0.9	0,5.1	1 / 84	1.2	0, 6.5
55+ y	7 / 103	6.8	2.8, 13.5	0 / 32	0		2 / 107	1.9	0.2, 6.6
Total	46 / 494	9.3	6.9, 12.2	1 / 504	0.2	0,1.1	6 / 499	1.2	0.4, 2.6
Age-standardized prevalence (local population)		13.1	8.8, 17.4		0.9	0, 2.0		0.4	0, 1.3

*n/N number of positive cultures divided by the number of stool samples cultured

### Serotype distribution and antimicrobial susceptibility patterns

Out of the 53 NTS isolates, 5 were *S*. Enteritidis, one was *S*. Typhimurium and the remaining 47 could not be typed fully by the anti-sera available (Table 3). Of the *S*. Enteritidis serotypes, 3 were in infants, 1 in a child 12–59 months, and 1 in an adult 15–54 years, while the single isolate of *S*. Typhimurium was in an adult >55 years. *S*. Typhi was not isolated in any of the sites. The distribution of *S*. Enteritidis and *S*. Typhimurium, did not vary by site, however, the prevalence of the other serotypes was higher in Kilifi (43/494, 8.7%), than in Nairobi (1/504, 0.2%) and Siaya (3/499, 0.6%), p<0.001. All the 53 NTS isolates were susceptible to the antibiotics tested in the panel.

**Table 3:** Serotype distribution of NTS isolates by site, Kenya, (2016–17).

	Kilifi	Nairobi	Siaya	Total
*S*. Enteritidis	3	0	2	5
*S*. Typhimurium	0	0	1	1
Other Group D	3	0	0	3
Other Group B	11	0	1	12
Other serotypes	29	1	2	32
**Total**	**46**	**1**	**6**	**53**

### Risk factors associated with fecal carriage of NTS

The distribution of risk factors among the participants varied by site (Table 1). The prevalence of anemia (hemoglobin <10g/dl) was 20% (58/290) in Kilifi, 7% (31/433) in Nairobi and 9% (20/277) in Siaya. Prevalence of asymptomatic *P*. *falciparum* infection was 8% (23/307) in Kilifi, 3% (11/443) in Nairobi and 28% (139/489) in Siaya. The prevalence of acute malnutrition (MUAC <12.5cm) among children aged less than 5 years was 16% (34/209) in Kilifi, 23% (54/233) in Nairobi and 4% (8/200) in Siaya. The distribution of animals kept also differed by site: there were more animals kept in Siaya than in any other site, but chicken farming was practiced across all sites (Table 1). As expected, more animals were kept among participants in the rural areas (Kilifi and Siaya) than in the urban slum area of Mukuru.

We did not include data from Nairobi in this analysis because there was only one positive culture among the 504 samples tested. In the univariate analysis of risk factors for prevalent NTS carriage, anemia (hemoglobin <10g/dl), recent malaria and malnutrition were not associated with NTS carriage (S1 Table). The risk of carriage was higher among participants who used a shared basin for washing hands than among those who washed separately (Prevalence Ratio 2.7, 95% CI 1.6, 4.7), but was not associated with any of the other WASH-related exposures assessed. Ownership of cattle and sheep was associated with a reduced risk of carriage of NTS (S1 Table). In addition, contact with cattle, goats, sheep or chickens was also associated with a reduced risk of carriage of NTS (S1 Table).

In the multivariable model including age and site as covariates, none of the risk factors that were significant on univariate analysis were associated with fecal carriage of NTS.

## Discussion

In this study, the prevalence of NTS carriage varied very markedly by site from 0.4% and 0.9% in Nairobi and Siaya to 13.1% in Kilifi. Overall, serotypes commonly associated with invasive disease accounted for only 6 of 53 carriage isolates and the high prevalence in Kilifi may have been related to the presence of serotypes other than *S*. Typhimurium/*S*. Enteritidis. Carriage prevalence in Kilifi was highest in young/middle aged adults and was not associated with sex. We observed no *in vitro* resistance to commonly used antimicrobials tested against our NTS isolates.

The observed carriage prevalence in Kilifi (13.1% all ages and 6.4% in <5y) is higher than that reported in previous population-based studies on fecal carriage of NTS among children and adults across several sites in Africa. For example, carriage prevalence across all ages was 1.0% in Dakar, Senegal, 2.4% in Bissau, Guinea-Bissau and 3.4% in Kifua, Democratic Republic of Congo (DRC) [25,26] while among children aged <5 years in the Global Enteric Multicenter Study (GEMS), the observed prevalence was 1.3% in Basse, The Gambia, 0% in Bamako, Mali and 0.1% in Manhica, Mozambique. However, the observed prevalence (1.5%, 95%CI 0.3–4.3) in Siaya among children aged <5 years in our study is comparable to that observed from GEMS study at the same site (3.2%, 2.5–4.1) [27].

The majority of the invasive serotypes isolated across Africa are *S*. Typhimurium or *S*. Enteritidis [28] though these serotypes represented only 11% (6/53) of the carried isolates in our study. In Senegal, Guinea-Bissau, DRC, The Gambia, Mozambique and Siaya (GEMS), these pathogenic serotypes were only detected in The Gambia (9/38, 24%) and Siaya (25/59, 41%) of all carriage serotypes isolated. The invasiveness of a given serotype can be expressed as the proportion of the serotype among invasive isolates divided by the proportion among carriage isolates [29]. In a previous study in Kilifi, these two pathogenic serotypes were responsible for 89% (312/351) of iNTS disease [17], yet account for only 6.5% (3/46) of carriage in this study; and in Siaya they were responsible for 98% (59/60) of iNTS disease in a previous study [30] and 50% (3/6) carriage in this study. Although estimation of relative invasive index is beyond the scope of this analysis (which included only carriage isolates), our findings suggest that these serotypes are more common among invasive isolates than among carriage isolates, and that the prevalence may vary across different settings. The outcome of an infection with *Salmonella* serotype depends on host susceptibility [7,31], infective dose [32] and pathogen virulence [33–35]. Furthermore, within-serotype differences in invasiveness have been demonstrated among different sequence types of *S*. Typhimurium [36], suggesting that the broad serotype classification may not capture all the attributes of invasiveness; classifications based on genotype may be more useful [37].

The antibiograms of the 53 isolates showed a full susceptibility profile, which is in marked contrast to the multi-drug resistant profiles seen in other NTS isolates [7], especially among invasive *S*. Typhimurium serotypes suggesting that resistance could be linked to virulence. In other studies, the same resistance profiles have been observed in asymptomatic and symptomatic isolates [27,38–41], suggesting that invasive disease may arise out of the pool of carried strains. However, the transmission of isolates causing asymptomatic carriage and of isolates causing iNTS may also differ and the relationship between carriage of antimicrobial resistant strains and the occurrence of drug-resistant invasive disease is not clearly understood.

Within country differences in the frequency of iNTS disease have been documented, and have mostly been attributed to differences in urbanization, agricultural practices and the presence of risk factors for invasive diseases such as malaria and HIV [19,42]. In this study, the prevalence of recent malaria was higher in Siaya (28%) than in Kilifi (7%) or Nairobi (2%). There were geographical differences in the occurrence of other host risk factors such as undernutrition in children but none of these were associated with carriage. Factors affecting the prevalence of iNTS may differ from those associated with NTS carriage. The apparent geographical differences may be explained by seasonality in occurrence of iNTS disease, since the sampling in the sites was done at different calendar times and seasons. Given the cross-sectional nature of the study, we could not assess the seasonal changes in NTS carriage. We could also not estimate how many of the cases of NTS carriage were asymptomatic or preceded or followed an invasive episode, though 18% of the participants (51% of whom were under 5y) had a febrile illness and 8% (67% of whom were under 5y) had a diarrhea episode in the 2 weeks prior to enrolment in the study. None of these risk factors when analyzed was associated with carriage.

Age-specific carriage prevalence observed in this study were similar to observations from Guinea-Bissau [25]; notably high carriage prevalence among adults and older children and low carriage prevalence among children aged <5 years. This could be explained by age-related differences in the acquisition of infection and persistence of infection. As NTS is a fecal-oral pathogen, neonates are exposed to it during vaginal delivery [43] but, subsequently, due to limited mobility and breastfeeding, their interactions with environmental sources of NTS in infancy are limited. On the other hand, older children and adults interact with a wider range of sources of NTS through the food supply such as animal meat and products such as dairy and eggs, and both raw and cooked vegetables, which are likely sources and may lead to colonization. Previous studies report a median duration of carriage of 7 weeks in children aged <5 years and 3–4 weeks in older children and adults [44]. Given that carriage prevalence rises with age in children, this implies that acquisition rates rise even more steeply with age [45].

This study was limited by the low sensitivity of stool culture for recovery of NTS. Also, given the cross-sectional nature of the sampling and the intermittent shedding of NTS among carriers, the estimates of prevalence in this study are likely to be underestimates of the true prevalence. Due to the low yield, we could not assess the relative prevalence of *S*. Typhimurium (1 isolate) to *S*. Enteritidis (5 isolates) recovered in this study. The current ratio does not reflect the known distribution of the two serotypes in Kenya (1:1). Use of multiple samples per time point [26] and use of direct-stool PCR with enrichment [46] may have improved the yield of *Salmonella*. We also lacked full serotype and genotype data of the isolates, and therefore could not comment on the invasiveness of all the isolates identified nor characterize the sequence types of interest such as *S*. Typhimurium ST313 and the different clades of *S*. Enteritidis.

Despite these limitations, this study provides comparative estimates of the prevalence of NTS carriage across different geographical locations in Kenya. Kilifi, the area with the lowest incidence of iNTS disease [17,18], had the highest prevalence of carriage (13%), while Siaya and Nairobi, which are areas of high and moderate disease incidence [19], had low carriage prevalence (0.9%, 0.2%). The observation suggests that diverse carriage strains, with lower invasive potential, could have a protective advantage if they induce a degree of cross-reactive immunity among the carriers. Further characterization of the isolates recovered, including sequencing, could enable identification of common antibody targets that might support this hypothesis.

## Supporting information

S1 Table Risk factors associated with fecal carriage of NTS in Kilifi. (DOCX)
