# Supplementary material for: The epidemiology of fecal carriage of nontyphoidal Salmonella among healthy children and adults in three sites in Kenya
Source: PLoS Negl Trop Dis. 2023 Oct 26;17(10):e0011716. doi: 10.1371/journal.pntd.0011716 (PMC10629669; doi:10.1371/journal.pntd.0011716)
Supplement: S1 Table — (DOCX) [file pntd.0011716.s001.docx]

Supplementary Table: Risk factors associated with fecal carriage of NTS in Kilifi and Siaya, Kenya, 2016-17.

|  | **Prevalence in exposed** | | **Prevalence in unexposed** | | **Prevalence ratio** | | **Adjusted prevalence ratio** | |
| --- | --- | --- | --- | --- | --- | --- | --- | --- |
| **Variable** | **n/N** | **%** | **n/N** | **%** | **ratio** | **95% CI** | **ratio** | **95% CI** |
| ***Host Factors*** |  |  |  |  |  |  |  |  |
| Sex, female | 33/556 | 5.9 | 19/437 | 4.4 | 1.4 | (0.8,2.4) |  |  |
| Recent malaria | 4/162 | 2.5 | 31/634 | 4.9 | 0.5 | (0.2,1.4) |  |  |
| Underweight (WAZ <-2)^†^ | 2/46 | 4.4 | 14/345 | 4.1 | 1.1 | (0.3,4.6) |  |  |
| Wasting (WHZ <-2)^†^ | 3/26 | 11.5 | 13/365 | 3.6 | 3.2 | (0.9,10.7) |  |  |
| Stunting (HAZ <-2)^†^ | 4/92 | 4.4 | 13/318 | 4.1 | 1.1 | (0.4,3.2) |  |  |
| Acute malnutrition (MUAC<12.5)^†^ | 4/42 | 9.5 | 13/367 | 3.5 | 2.7 | (0.9,7.9) |  |  |
| Anemia (Hb<10) | 3/78 | 3.9 | 27/450 | 6.0 | 0.6 | (0.2,2.1) |  |  |
| ***Clinical illness in last 2 weeks*** |  |  |  |  |  |  |  |  |
| Admitted to hospital | 0/14 | 0 | 47/943 | 5.0 | - |  |  |  |
| Diarrhea | 1/60 | 1.7 | 46/897 | 5.1 | 0.3 | (0.04,2.3) |  |  |
| Fever | 8/213 | 3.8 | 39/744 | 5.2 | 0.7 | (0.3,1.5) |  |  |
| ***Household illness in last 2 weeks*** | | |  |  |  |  |  |  |
| Household member had diarrhea | 3/56 | 5.4 | 44/901 | 4.9 | 1.1 | (0.4,3.4) |  |  |
| Household member had fever | 10/200 | 5.0 | 37/757 | 4.9 | 1.0 | (0.5,2.0) |  |  |
| ***WASH Practices*** |  |  |  |  |  |  |  |  |
| Source of drinking water |  |  |  |  |  |  |  |  |
| Piped water | 8/98 | 8.2 |  |  | Ref |  |  |  |
| Public tap/vendors | 24/450 | 5.3 |  |  | 0.7 | (0.3,1.4) |  |  |
| River/Stream/Lake | 1/78 | 1.3 |  |  | 1.6 | (0.0,1.2) |  |  |
| Well/Dam/Borehole | 14/331 | 4.2 |  |  | 0.5 | (0.2,1.2) |  |  |
| Uses soap to wash hands | 38/814 | 4.7 | 9/143 | 6.3 | 0.7 | (0.4,1.5) |  |  |
| Uses shared wash basin | 23/250 | 9.2 | 24/707 | 3.4 | 2.7 | (1.6,4.7) | 1.4 | (0.8,2.5) |
| Type of toilet |  |  |  |  |  |  |  |  |
| Modern with/without flush | 5/52 | 9.6 |  |  | Ref |  |  |  |
| Pit latrine | 39/873 | 4.5 |  |  | 0.5 | (0.2,1.1) |  |  |
| None/Open defecation | 1/20 | 5.0 |  |  | 0.5 | (0.1,4.2) |  |  |
| Other | 2/12 | 16.7 |  |  | 1.7 | (0.4,7.9) |  |  |
| ***Animal Ownership*** |  |  |  |  |  |  |  |  |
| Cattle | 9/366 | 2.5 | 38/591 | 6.4 | 0.4 | (0.2,0.8) | 1.5 | (0.6,4.0) |
| Sheep | 1/146 | 0.7 | 46/811 | 5.7 | 0.1 | (0.0,0.9) | 0.5 | (0.1,3.6) |
| Goats | 10/323 | 3.1 | 37/634 | 5.8 | 0.5 | (0.3,1.1) |  |  |
| Pigs | 0/4 | 0 | 47/953 | 4.9 | - |  |  |  |
| Chicken | 30/708 | 4.2 | 17/249 | 6.8 | 0.6 | (0.3,1.1) |  |  |
| ***Animal Contacts*** |  |  |  |  |  |  |  |  |
| Cattle | 7/439 | 1.6 | 40/518 | 7.7 | 0.2 | (0.1,0.4) | 0.7 | (0.2,2.6) |
| Sheep | 2/280 | 0.7 | 45/677 | 6.7 | 0.1 | (0.0,0.4) | 0.6 | (0.1,2.7) |
| Goats | 7/399 | 1.8 | 40/558 | 7.2 | 0.2 | (0.1,0.5) | 0.8 | (0.3,1.9) |
| Pigs | 0/14 | 0 | 47/943 | 4.9 | - |  |  |  |
| Chicken | 17/642 | 2.7 | 30/315 | 9.5 | 0.3 | (0.2,0.5) | 0.7 | (0.3,1.3) |

^†^Prevalence of malnutrition estimated only among children <5 years old

Average household size among carriers was 5.3 (SD 2.5) while that of non-carriers was 5.6 (SD 2.6)

Adjusted prevalence ratio from multivariable analysis. Only univariate associations with p<0.05 were included in multivariable analysis.
